# Profiling the human response to physical exercise: a computational strategy for the identification and kinetic analysis of metabolic biomarkers

**DOI:** 10.1186/2043-9113-1-34

**Published:** 2011-12-19

**Authors:** Michael Netzer, Klaus M Weinberger, Michael Handler, Michael Seger, Xiaocong Fang, Karl G Kugler, Armin Graber, Christian Baumgartner

**Affiliations:** 1 Research Group for Clinical Bioinformatics, Institute of Electrical, Electronic and Bioengineering, UMIT, 6060 Hall in Tirol, Austria; 2 Institute for Bioinformatics and Translational Research, UMIT, 6060 Hall in Tirol, Austria; 3 Biocrates Life Sciences AG, Innrain 66/2, 6020 Innsbruck, Austria; 4 Bavarian Nordic GmbH, Fraunhoferstr. 13, 82152 Martinsried, Munich, Germany; 5 Zhongshan Hospital, Fudan University, 200032 Xuhui District, Shanghai, China

## Abstract

**Background:**

In metabolomics, biomarker discovery is a highly data driven process and requires sophisticated computational methods for the search and prioritization of novel and unforeseen biomarkers in data, typically gathered in preclinical or clinical studies. In particular, the discovery of biomarker candidates from longitudinal cohort studies is crucial for kinetic analysis to better understand complex metabolic processes in the organism during physical activity.

**Findings:**

In this work we introduce a novel computational strategy that allows to identify and study kinetic changes of putative biomarkers using targeted MS/MS profiling data from time series cohort studies or other cross-over designs. We propose a prioritization model with the objective of classifying biomarker candidates according to their discriminatory ability and couple this discovery step with a novel network-based approach to visualize, review and interpret key metabolites and their dynamic interactions within the network. The application of our method on longitudinal stress test data revealed a panel of metabolic signatures, i.e., lactate, alanine, glycine and the short-chain fatty acids C2 and C3 in trained and physically fit persons during bicycle exercise.

**Conclusions:**

We propose a new computational method for the discovery of new signatures in dynamic metabolic profiling data which revealed known and unexpected candidate biomarkers in physical activity. Many of them could be verified and confirmed by literature. Our computational approach is freely available as R package termed BiomarkeR under LGPL via CRAN http://cran.r-project.org/web/packages/BiomarkeR/.

## Introduction

In metabolomics the bioinformatics-driven search for highly-discriminatory biomarker candidates has become a key task in the biomarker discovery process with the objective of introducing novel biomarkers aiding in diagnosis or therapeutic management [1-4].

A wide spectrum of feature selection methods including filter, wrapper or embedded algorithms is available for the identification of significant features in biomedical datasets [5-9]. In particular filter algorithms calculate a measure (score), allowing to rank and prioritize putative biomarker candidates according to their predictive value [8]. However, research is still needed to provide bioinformatics methods for the scientific community that address paired/dependent test hypotheses or time series studies. In addition, the quantitative analysis of networks has increasingly become an important technique for the biological interpretation of changes in disease-associated metabolic pathways, allowing the study of interconnectivity, interaction or correlation among analytes. For this type of analysis, different types of topological graph descriptors (e.g., parametric or partition-based entropy measures) can be used to analyze such complex biological networks [10,11].

In this short report we propose a new computational strategy that identifies metabolic biomarker candidates according to their discriminatory ability from dependent samples, and we review and interpret them using a network-based approach. For the biomarker identification and prioritization step we apply a recently published filter algorithm, named *Biomarker Identifier *(BI), which calculates a score measure for every analyte, representing the discriminatory ability in terms of the product of sensitivity and specificity, and in an analogous way for paired samples [12]. After BI prioritization we apply a new method to infer a network from the data by calculating analyte ratios, representing interactions of analyte pairs in the network. This discovery step aims at verifying metabolites selected from the first step, and reviewing identified highly discriminatory analyte pairs according to their connectivity strength within the network. This connectivity network permits scientists to review single and multiple pathway reactions, e.g., by mapping this information on biochemical network databases like KEGG [13] for identifying functional changes or abnormalities in human metabolism. Finally, we demonstrate results of this approach using targeted MS/MS profiling data for the search of metabolic signatures in physically fit persons during bicycle exercise, yielding known and partly unexpected interactions among analytes of physical activity.

## Computational strategy

### Step 1: Feature ranking and prioritization model

We apply the so-called BI model for selecting and prioritizing analytes into classes of weak, moderate and strong predictors, addressing both dependent and independent test hypotheses. In this work we focus in particular on metabolites changing over time for a given cohort (paired or dependent sample). The paired BI(pBI) is thus defined as [12]:

(1) $$pBI=\lambda \cdot DA^{*}\cdot \sqrt{\left|\frac{\Delta_{change}}{CV}\right|}.\;\text{sign(}\Delta_{change}),$$

(2) $$\Delta_{change}=\left\{\begin{array}{cc} \Delta & \text{if}\;\Delta \geq 1\\ -\frac{1}{\Delta } & \text{else} \end{array}\right)$$

where *λ *is a scaling factor, DA* is a discriminance measure defined as percent change of metabolite levels in one direction versus baseline and Δ*_change _*represents the median percent change. *CV *is the coefficient of variation and is set to 1 if *CV *> 1 by default to consider solely data distributions with smaller variance [12].

### Step 2: Network inference

By definition, a network *G *is defined as a set of vertices *V *which are connected by edges *E*: *G *= (*V*, *E*) [14]. Inferring the network includes three steps: (i) calculating all ratios *R *between metabolites *M *which represent chemical interactions, where $r_{ij}=|\log_{2}\left(\frac{m_{i}}{m_{j}}\right)|$ with *i *>*j*, and *m *∈ *M*, *r *∈ *R*. The logarithm induces symmetry of the ratios and their reciprocals, respectively. Note that by definition the metabolite concentrations must be positive (*m *≥ 0); (ii) computing pBI scores *s_ij _*, *s *∈ *S *on the logarithmic ratios *R *and (iii) constructing a graph *G *with:

(3) $$G_{ij}=\left\{\begin{array}{cc} 1 & \text{if}\;|s_{ij}|>\tau \\ 0 & \text{else}, \end{array}\right)$$

for *i*, *j *∈ 1, ..., |*M*|. A ratio *r *∈ *R *is designated as a putative pathway reaction of the form A → B, where a reactant A is metabolized into a product B via single or multiple reaction paths. To consider significant predictor pairs in the network the threshold *τ *has been evaluated using controlled simulated data in form of *D*(*D *~ *N*(10, 1), see "Additional file 1") as proposed by Guo et al. [15]. Next, we inferred the network for different values for *τ *and used vertices (metabolites) with at least one edge (i.e., degree > 0) as input for classification and calculated the mean accuracy of the classifier using 10-fold cross-validation (see "Additional file 1").

In contrast to a static network, typically constructed from data of independent case/control studies, a kinetic network can be inferred on single analytes or analyte pairs (as done in this work) with changes in levels greater than the fixed threshold *τ *at timepoint *t_x _*vs. baseline (*t*_0 _), representing the dynamics of circulating metabolites over time.

Coupling step 1 with step 2 of our discovery strategy allows for verifying preselected metabolites of step 1 as highly connected vertices (hubs) in the network in step 2. Note that a high degree of interconnectivity of a hub (i) represents analogously a high discriminatory and predictive value of this hub, and (ii) embedded in a network of pathway reactions a key role of normal or abnormal metabolism.

Our computational approach is freely available as R package termed BiomarkeR under LGPL via CRAN http://cran.r-project.org/web/packages/BiomarkeR/. We chose to implement our method in R because of the broad abundance of this programming language in the bioinformatics community and its open source nature. Additionally, there is a multitude of packages available for the handling and analysis of network-based data (e.g., igraph[16], QuACN[11] or BioNet[17]).

### Bicycle stress test

We here present a new computational method for the search for stress biomarkers in physically fit persons using targeted MS/MS profiling technology. Using this approach 60 analytes were identified by applying characteristic mass transitions in multiple reaction monitoring, precursor and neutral loss scans, and quantified using internal standards added to the samples [18].

Briefly, metabolite profiling of blood samples of a total of 30 active and physically fit individuals (22 males, 8 females) with a mean age of 38.33 ± 7.16 years and mean body mass index 23.88 ± 2.50 kg/m^2 ^was carried out. The used data represents analyses of residual material of a standard sport-physical examination where each proband had to bicycle on an exercise bike for increasing steps of Watt (W) levels (each step 25 W) until the individual's maximum capacity was reached.

Capillary blood samples were obtained from the ear lobe before starting the exercise (at rest, *t*_0 _), and at all Watt levels up to the individual's maximum performance (*t_max _*). A total of 60 metabolites (lactate, amino acids and acyl carnitines) were measured in absolute concentration values (*μ*mol/L) [19]. All individuals gave written informed consent to the attending physician.

## Results and discussion

**Figure 1:**
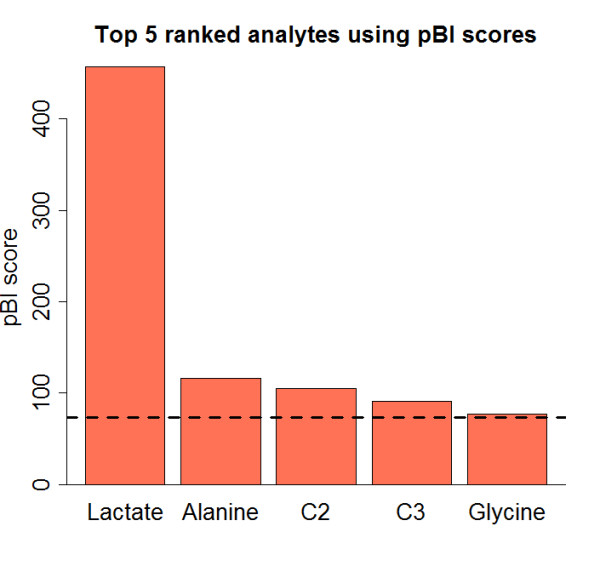
**pBI scores**. pBI scores of top ranked analytes lactate, alanine, C2, C3 and glycine showing increased levels when comparing subjects at rest vs. individual maximum. Dashed lines indicate the score cut-off for strong predictors (|pBI| > 73), as defined in [12]. images/pBIScores.png.

**Figure 2:**
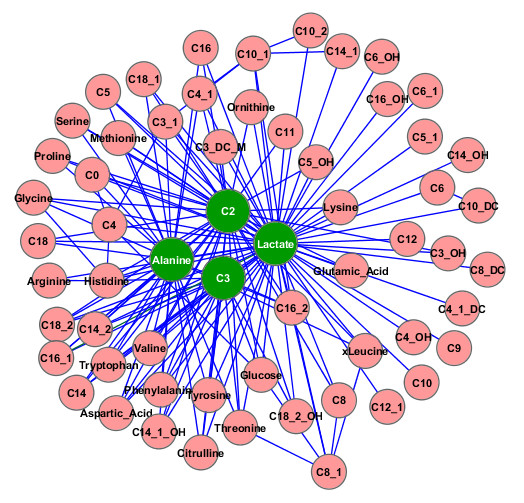
**Inferred network**. The resulting kinetic network (*τ *= 73) exhibits lactate, alanine, C2 and C3 as major hubs (green color) in the center of the network with the highest degree of connectivity. Note that an edge between metabolite *m_i _*and *m_j _*indicates a pBI value of the absolute logarithmic ratio of *m_i _*and *m_j _*greater than *τ *(e.g., for the edge between C2 and C16 |*pBI*(|log_2 _(*C*2/*C*16)|)| > 73). Identified key metabolites during physical activity could also be validated by literature [22,23,32]. The number following the underscore of acyl-carnitines symbols indicates the number of double binds of the carbon-carbon bonds (e.g., for C10_1 the number of double binds is 1). The network is visualized using Cytoscape [33]. images/pbiGraph.png.

Using our computational approach the pBI priority model was applied as first step to preselect key metabolites from the measured pool of 60 analytes by computing scores at timepoint *t*_0 _(at rest) vs. *t_max _*(at individual maximum performance). Figure 1 shows the pBI scores, exhibiting five metabolites (i.e., lactate, alanine, glycine and the two short-chain acyl carnitines C2 and C3) categorized as strong predictors. Figure 2 demonstrates the corresponding dynamic network for *τ *= 73 again with lactate, alanine, C2 and C3 as major hubs, and now in the role as representatives of a panel of reaction pairs, coinciding with the univariate pBI metabolite ranking. Interestingly, using the controlled simulated data a threshold of *τ *= 73, which corresponds to the cut-off score for a strong predictor as defined in [12], led to the maximum mean accuracy using a K-nearest-neighbor classifier [20], outperforming also commonly used correlation-networks [21] (see "Additional file 1").

To validate our findings a literature review and analysis of related KEGG pathways were performed. Among the total of 60 detected metabolites, our analysis revealed 5 key metabolites (lactate, alanine, glycine, and the two short-chain acyl carnitines C2 and C3) associated with physical exercise. These results are consistent with previous reports [19,22,23]. As is well known, anaerobic glycolysis is the main way for energy supply during exercise workout. Lactate is a major end product of the metabolism of glucose through the glycolytic pathway [23]. The skeletal muscle is the main organ producing large amounts of lactate. Typically, the production of lactate is greatly increased during exercise via the oxidative metabolism [23]. In our work, we detected a pBI score for lactate greater than 400 which represents a strong correlation with physical exercise. The known biochemical or physiologic effects of carnitine suggest that supplementation of carnitine may improve exercise performance [24,25]. First, carnitine is required for mitochondrial fatty acid oxidation, which would permit glucose utilization to decrease, and thus preserve muscle glycogen content and ensure maximal rates of oxidative ATP production [24,26]. Second, generation of acetylcarnitine would potentially decrease acetyl-CoA content, relieving inhibition of pyruvate dehydrogenase and decrease the production of lactate [23,24,27]. All of these can potentially improve physical performance during high-intensity exercise. Besides, recent studies demonstrated that short term administration of glycine propionyl-L-carnitine (GPLC) significantly elevates levels of nitric oxide metabolites at rest and in response to reactive hyperemia [28-30], and can also enhance exercise performance in healthy, trained individuals [28]. Carnosine is synthesized in skeletal muscle from L-histidine and A-alanine amino acids [22]. One important physiological role of carnosine is the maintenance of acid-base homeostasis [22,31]. Studies have shown that supplementation with A-alanine or exercise can increase muscle carnosine content and therefore total muscle buffer capacity with the potential to cause improvements in physical exercise [19,22].

Overall, more than 20 pathways were revealed. We selected the most related pathways which include at least 2 metabolites identified by our coupled 2-step discovery step (Table 1). It shows that the citrate cycle (TCA cycle), multiple amino acid and fatty acid metabolisms are greatly activated in physical exercise.

**Table 1:** KEGG Pathways

KEGG ID	KEGG pathway	Related metabolites
Map00330	Arginine and proline metabolism	Alanine, aspartate, ornithine, proline, citrulline, arginine, glutamic acid, lysine, methionine, glycine
Map00020*	Citrate cycle	Glycose, leucine, valine, arginine, proline, tyrosine, alanine, aspartate, glutamic acid
Map00260	Glycine, serine and threonine metabolism	Serine, glycine, threonine, valine, aspartate, lysine, proline, methionine, leucine, arginine
Map00250	Alanine, aspartate and glutamate metabolism	aspartate, arginine, histidine, glutamic acid, alanine, proline, lysine, serine, glycine, glucose
Map00620*	Pyruvate metabolism	Glycine, lactate, serine, leucine, lysine, valine, threonine
Map00640	Propanoate metabolism	Alanine, lactate, leucine, methionine, valine
Map00270	Cysteine and methionine	Methionine, threonine, alanine, aspartic acid, glycine, serine
Map00473	D-alanine metabolism	Alanine, aspartate, glutamate, glucose, ornithine
Map00410	*β*-alanine metabolism	Aspartate, alanine, histidine, lysine, arginine
Map00460	Cyano amino acid metabolism	Serine, glycine, alanine, glutamate, aspartate
Map00300	Lysine biosynthesis	Glycine, serine, threonine, lysine, alanine
Map00710	Carbon fixation in photosynthetic organisms	Tyrosine, phenylalanine, alanine, aspartate, glycose
Map00071*	Fatty acid metabolism	Multiple carnitines, alanine, aspartate

Identified KEGG pathways in which metabolites selected by the inferred network are shown. The asterisks (*) indicate metabolic pathways associated with physical exercise (see also section "Results and discussion").

## Conclusion

We have introduced a powerful tool for the search, prioritization and network analysis of putative biomarker candidates in metabolomic studies. Our 2-step approach has several benefits: 1) BI can be applied to dependent samples, calculating absolute scores for prioritizing biomarker candidates into classes of weak, moderate or strong predictors, and computes positive and negative scores, indicating whether the metabolites concentration is increased or decreased compared to its reference. 2) The proposed approach allows to review and interpret findings and thus aids in biochemical interpretation of (ab)normal metabolism by reviewing pathway reactions within the network.

Using our coupled 2-step discovery strategy, we were able to identify and confirm multiple metabolites, i.e., lactate, glycine, alanine, C2 and C3 that are closely associated with metabolism of physical activity [22,23,32].

## Competing interests

The authors declare that they have no competing interests.

## Authors' contributions

MN and CB designed and wrote the manuscript. KW and AG performed mass spectrometry analysis and joined in data interpretation. KK and MN drafted and developed the network inference method. XF performed pathway analysis and interpretation. MN, MH, MS and KK designed and implemented the BiomarkeR package. All authors have read and approved the final manuscript.

## Supplementary Material

Additional file 1 **Simulation results**. This PDF file contains an additional figure comparing mean accuracies for different thresholds using controlled simulated data and a K-nearest-neighbor as classifier. Click here for file
