# Supplementary material for: Profiling the human response to physical exercise: a computational strategy for the identification and kinetic analysis of metabolic biomarkers
Source: J Clin Bioinforma. 2011 Dec 19;1:34. doi: 10.1186/2043-9113-1-34 (PMC3320562; doi:10.1186/2043-9113-1-34)
Supplement: Additional file 1 — Simulation results. This PDF file contains an additional figure comparing mean accuracies for different thresholds using controlled simulated data and a K-nearest-neighbor as classifier. [file 2043-9113-1-34-S1.PDF]

Additional Files

Additional file 1 — Simulation results

In order to analyze the parameter τ we used controlled simulated data as proposed by Guo et al. [16]. In total the dataset comprised 100 features with 50 samples for each timepoint t_0 and t_1. 80 features showed no difference between the groups (effect size = 0), 10 showed maximal difference ("biomarker candidates") with an effect size 1, and the residual 10 features are correlated with ten randomly selected features out of the pool of 90. We created normal distributed data (N(10, 1)) with 50 samples for timepoint t_0. In particular, the data for timepoint t_1 were generated from the data at t_0 interfered with normally distributed noise (N(0, 0.1)).

The mean accuracies for $\tau = \{50, 60, 70, 73, 80, 90, 100\}$, using all vertices with a degree > 0 as input for classification, are depicted in Figure A1. For these experiments we used a K-nearest-neighbor as classifier. We set the number of neighbors to $k = 7$, demonstrating maximum classification accuracy after 10-fold cross-validation. The results revealed the highest accuracy of 0.91 for the thresholds τ between 73 and 80.

We also compared our results with a correlation network. Therefore, we inferred a Pearson correlation network ($\alpha = 0.05$) for each class and calculated the differences of vertex degrees (Δ_{deg}). Using all vertices with $|\Delta_{deg}| > 0$ as input for classification an accuracy of 0.6 was calculated, demonstrating significantly lower performance than our network approach.

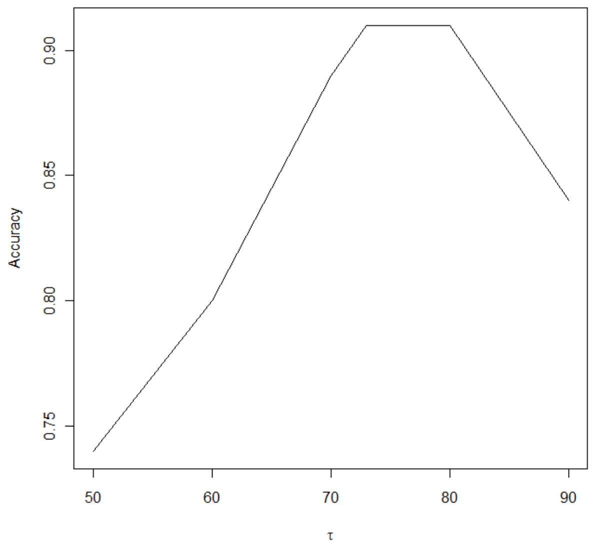

Figure A1: The mean accuracies for $\tau = \{50, 60, 70, 73, 80, 90, 100\}$.
